# Organoids as host models for infection biology – a review of methods

**DOI:** 10.1038/s12276-021-00629-4

**Published:** 2021-10-18

**Authors:** Carmen Aguilar, Marta Alves da Silva, Margarida Saraiva, Mastura Neyazi, I. Anna S. Olsson, Sina Bartfeld

**Affiliations:** 1 Research Centre for Infectious Diseases, Institute for Molecular Infection Biology, Julius Maximilians Universität Wuerzburg, Wuerzburg, Germany; 2 i3S - Instituto de Investigação e Inovação em Saúde, Universidade do Porto, Porto, Portugal; 3 IBMC- Instituto de Biologia Celular e Molecular, Universidade do Porto, Porto, Portugal

**Keywords:** Biological models, Adult stem cells

## Abstract

Infectious diseases are a major threat worldwide. With the alarming rise of antimicrobial resistance and emergence of new potential pathogens, a better understanding of the infection process is urgently needed. Over the last century, the development of in vitro and in vivo models has led to remarkable contributions to the current knowledge in the field of infection biology. However, applying recent advances in organoid culture technology to research infectious diseases is now taking the field to a higher level of complexity. Here, we describe the current methods available for the study of infectious diseases using organoid cultures.

## Infectious diseases: Mini-organ studies could facilitate personalized treatments

Using miniaturized, three-dimensional versions of organs and tissues to model infectious diseases could improve understanding of patient-specific responses and inform personalized therapies. Organoid cultures are generated from tissue-specific adult stem cells, and recreate some of the original cellular structure of a given organ or tissue. They also mimic the organ’s molecular mechanisms and responses. Sina Bartfeld at the Julius Maximilians Universität Würzburg, Germany, and coworkers reviewed recent research into organoid cultures for modeling infections and disease progression. Creating organoids from different individuals allows scientists to monitor how infections behave in unique hosts. Organoid studies have highlighted specific targets for initial infection by bacteria, identified host factors that influence disease outcomes, and clarified patient-specific responses to treatments. Future organoids should be more complex, incorporating immune and nerve cells, and even factoring in the microbiome.

## Introduction

### Why we need new models

**Fig. 1:**
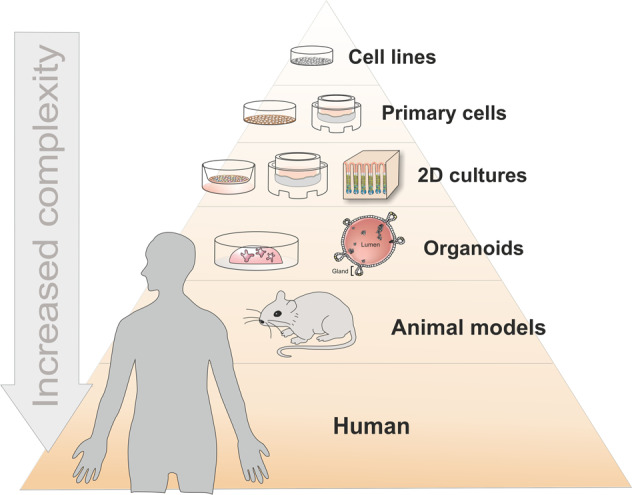
Complexity of current models available for the study of human infectious diseases. Cell lines are widely accessible and practical but lack epithelial complexity. Organoids and organoid-derived primary cell monolayers consist of primary nontransformed cells that can differentiate into different cell types. Animal models allow the study of host–pathogen interactions in whole organisms; however, pathogens are often highly adapted to their natural host, and thus animal studies have limitations in the study of human infectious diseases.

As the SARS-CoV-2 pandemic has illustrated all too clearly, infections are still a major threat worldwide. Laboratory model systems are key to reaching a mechanistic understanding of disease development. In the study of host–pathogen interactions, such model systems must rise to the challenge of modeling not only a single organism, i.e., the host, but also its interplay with microorganisms. Infecting microorganisms often initiate a host response, such as inflammation, and may even manipulate the host response to benefit their own survival, e.g., by repressing the inflammatory response. Evolution has thus yielded highly specialized and adapted pathogens that engage with the host to create their very specific local environment or niche. Scientists have used many different model systems, each with their specific advantages and each leading to important and valuable results (Fig. 1).

Given that epithelial cells are generally the first point of contact with a pathogen and first to respond to it, epithelial cell lines are one of the most commonly used cell models. Since the establishment of HeLa cells in the 1950s, a vast number of cell lines have been developed and have become workhorses in biomedical research, including epithelial, immune, endothelial, neural, and mesenchymal cell lines. They are cheap, easy to expand, and easy to handle, allowing convenient implementation and enabling the high-throughput screening of drugs and virulence factors, among others. However, they are frequently cancerous, transformed or genetically immortalized, and often fail to mimic important epithelial functions, such as polarization, barrier formation, and cell differentiation. They are usually grown as 2D flat monolayers that contain only one cell type and lack the 3D structural characteristics of tissue, as well as the interactions with the extracellular matrix (ECM) and other cell types, such as immune cells, endothelial cells, or neurons. Even when grown in 3D structures, only one cell type from the epithelium is present, providing a reductionist model that is most suited to facilitating the understanding of a given specific cellular mechanism.

Primary cell cultures and explants can overcome some of these drawbacks. They are isolated directly from the specific tissue and are therefore not transformed and can recapitulate tissue functions in vitro. However, their low expansion potential and dependence on significant amounts of fresh tissue prevent their wide use in the study of human cells.

Animal models allow the study of infectious diseases with higher complexity, considering the interconnections among different organs and tissues involved, such as the epithelium and immune cells. A wide selection of animal models has been adapted for studying host–pathogen interactions, ranging from low-complexity models, such as the nematode *Caenorhabditis elegans*, to models with high complexity and similarity to humans, such as nonhuman primate models^1^. For a number of practical reasons, including the availability of genetic tools, mice are the most widely used model of experimental infection. However, the limitations of murine models are particularly evident in drug development, where more than 80% of therapeutic treatments that are effective and safe in animal models fail to succeed in human trials^2^. Furthermore, some pathogens have a narrow tropism for humans, which limits the use of mice and other animal models, although the development of humanized mouse models may help to overcome some of these limitations^3^. For example, the newly described SARS-CoV-2 virus does not cause disease in wild-type mice but does so in mice engineered to express the human receptor ACE2^4^.

Whereas higher complexity is desirable for investigating the events taking place at the whole-organism level, those models are not scalable to large studies, such as drug screening studies. Therefore, cell line-based reductionist models are useful for addressing specific questions, despite the neglect of other host players in host–pathogen crosstalk. The search for new, advanced model systems that allow us to deepen our understanding of host–pathogen interactions has progressed in recent years with the development of organoid technology. This powerful new method for culturing primary cells has provided a promising and versatile approach for numerous fields, ranging from infection to tissue development and regeneration to cancer^1,5–7^. Here, we review the different approaches for using organoids as models for infection biology.

## Methods for using organoids to study infection

### Organoids

**Fig. 2:**
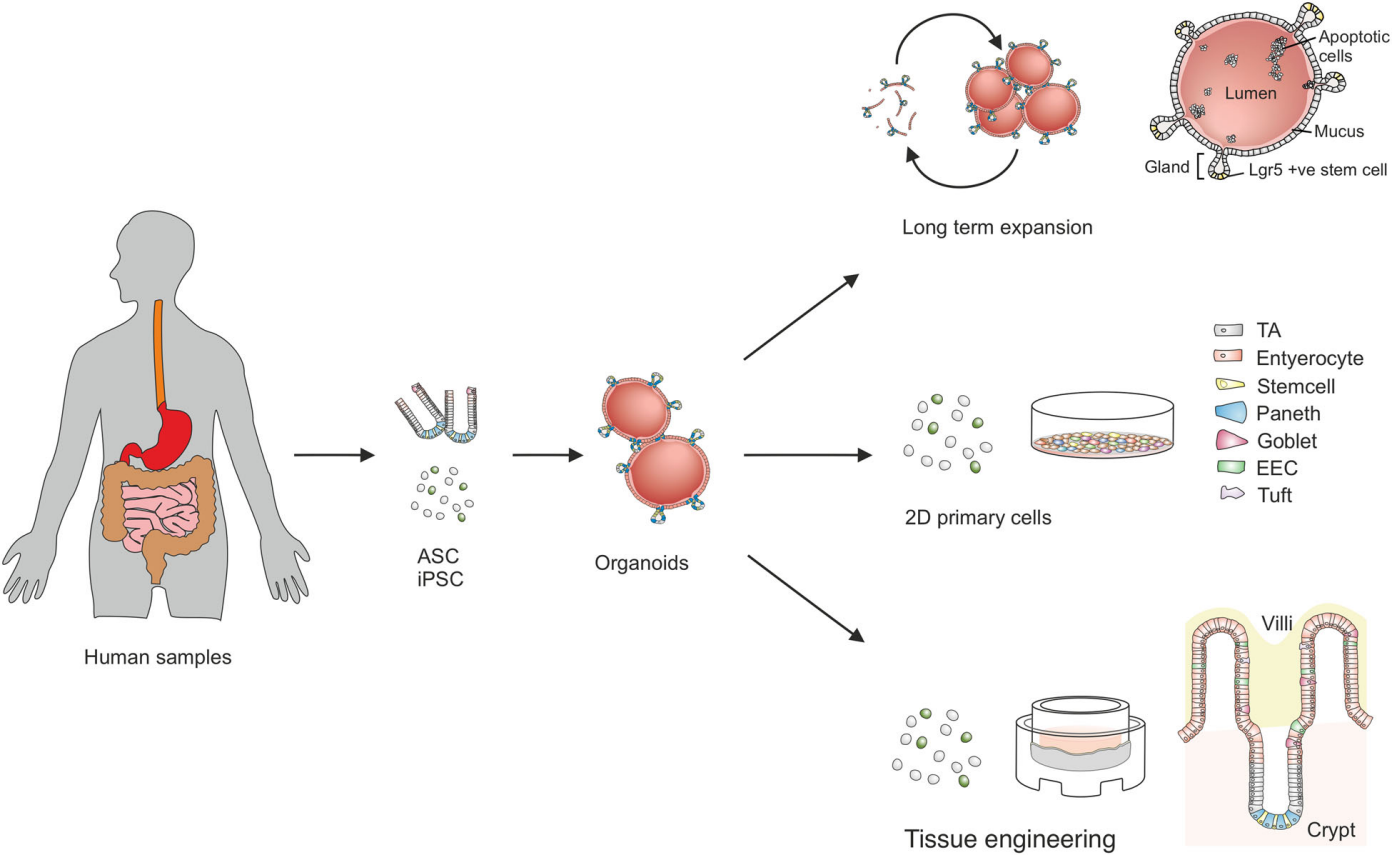
Organoids and organoid-derived models. Organoids are derived from tissue-specific adult stem cells (ASCs) or pluripotent stem cells (iPSCs). The cells differentiate into different cell types, which self-organize into domains and thus resemble the native tissue architecture, yielding reductionist models of organs. ASCs can easily be maintained in culture for long-term expansion, seeded in 2D monolayers, and seeded in tissue engineering models, including scaffolds, providing more structural complexity to resemble the physiological microenvironment for cell growth and distribution.

Organoids fill the gap between monotypic cells and complex organisms. The term “organoid” has been used in the past to describe the histological characteristics of tumors or other tissues and generally meant “resembling an organ”. The stem cell field has, however, redefined this term to describe 3D cell cultures derived from stem cells, in which the cells self-organize and exhibit at least some of the functions that they would have in vivo^8^. The technology originated from parallel developments in the two major fields of stem cell research: adult stem cells^9^ and pluripotent stem cells^10^. Today, the two technologies cover an impressive range of organs that can be mimicked^1,5–7^. In organoid cultures, isolated stem cells are placed in a dome of ECM and provided with a specific cocktail of growth factors. To generate organoids from pluripotent stem cells, growth factors mimic embryonic development. As a result, these organoids harbor very different cell types, such as mesenchymal and epithelial cells, in the same organoid, and the organoids have a rather limited expansion potential. In contrast, to generate organoids from adult tissue-specific stem cells, the growth factors provided mimic tissue regeneration. The resulting organoids consist of pure epithelial cells, and many types of these organoids have been shown to have tremendous expansion potential with very high genetic stability^11,12^. In prototypes of adult stem cell-derived cultures for gastrointestinal organoids, cells grow to generate a single epithelial layer surrounding a central lumen. Cells show specific polarization, with the apical side facing the organoid lumen. The organoid can present invaginations, or buddings, of the cell layer that resemble the intestinal crypts or gastric glands. In addition, the growth factor cocktail can be modified to induce the differentiation of specific cell types of interest, for example, intestinal adsorptive cells versus secretory cells or gastric pit cells versus gland cells^9,13,14^. Importantly, because organoids are a source of expandable primary cells, their cells can also be used to establish other in vitro models, such as 2D monolayers or more advanced tissue engineering (TE) models (Fig. 2).

#### Starting questions

To study infection in organoids, three central questions should be considered: What is the natural site of the host–pathogen interaction; what is the natural target cell for infection; and which host factors could be crucial for determining the outcome of the infection? For example, in the gastrointestinal tract, an ingested pathogen reaches the lumen of the gastrointestinal tract and then interacts with the apical side of epithelial cells. In contrast, pathogens that disrupt the epithelial barrier or that are spread via immune cells can also reach the basolateral side of host cells. Regarding the target cells, some pathogens are known to exhibit very narrow cell-type tropisms, while others infect a range of cell types. For many pathogens, it is not yet known which cells can be infected. Last, organoids are generated from specific individuals and retain patient-specific characteristics^1,15^, such as expression profiles or treatment responses^16–18^. Therefore, it seems likely that infection studies in organoids could mirror observed patient specificity regarding susceptibility to infection or variation in the infectious disease outcome. While this enables questions regarding patient specificity in infection to be asked, it should also be kept in mind regarding the experimental setup, and experiments should be performed using organoid lines from at least three different patients. We suggest using these considerations as a starting point for selecting the appropriate method.

#### Microinjection of organoids

**Fig. 3:**
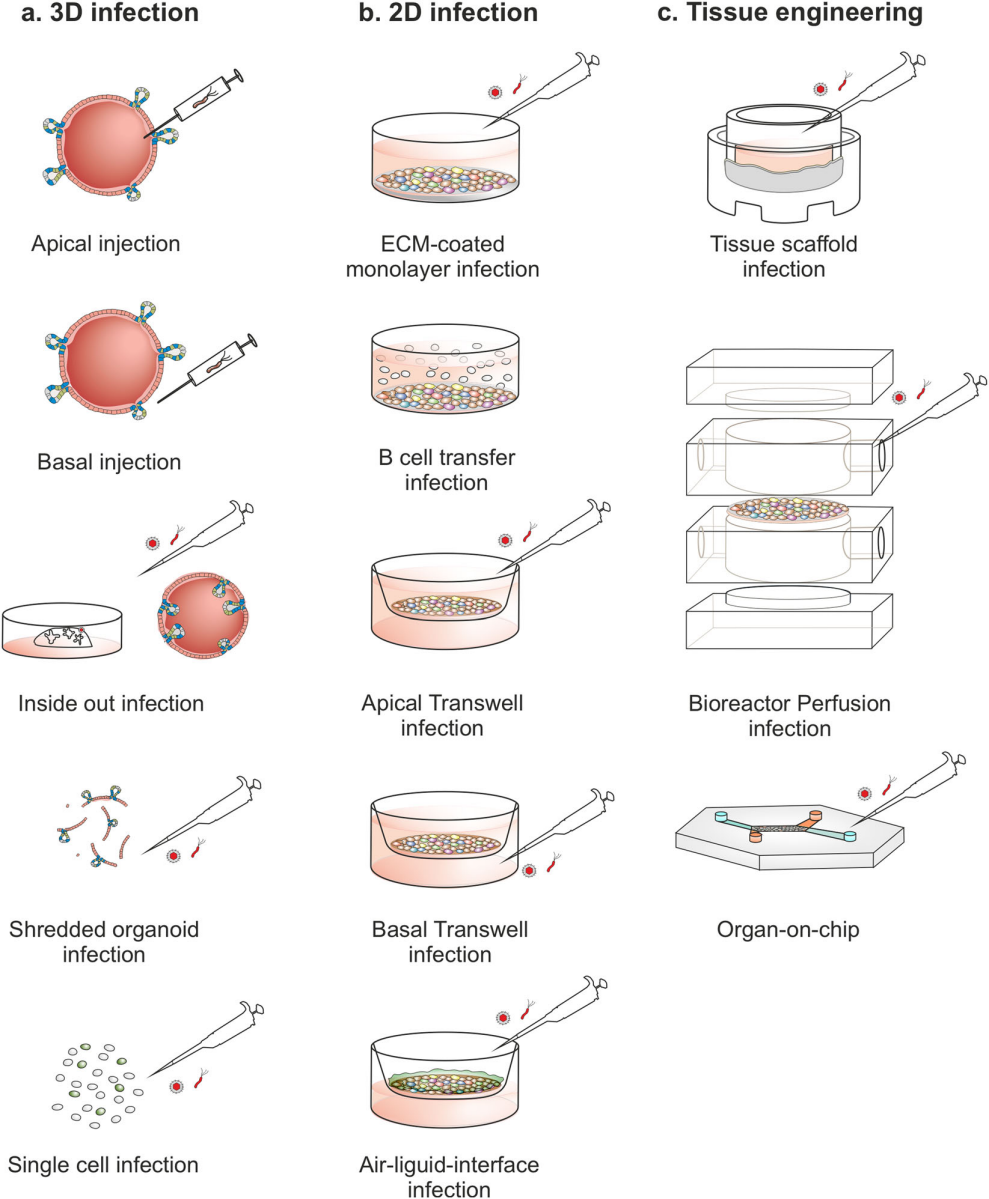
Methods using organoids to study infections. **a** Infection models using organoids: in their intact 3D structural form with apical or basolateral application of the infectious agent, inside-out infection, shredded organoids cocultured with pathogens and single-cell coculture infection. **b** Organoid-derived 2D monolayer infection models: primary cells with ECM coating, B cell transfer infection, apical or basolateral transwell infection. **c** Tissue engineering infection models seeded with organoid-derived primary cells: tissue scaffold infection, bioreactor perfusion infection, and organ-on-a-chip perfusion infection.

One of the first techniques used to infect organoids was microinjection of pathogens into the lumen, which allows them to interact with the apical side of host cells (Fig. 3a). Microinjection with bacteria, such as *Helicobacter pylori* (*H. pylori)*^13,19–22^, *Clostridioides difficile* (*C. difficile)*^23,24^, *Escherichia coli* (*E. coli*)^25,26^, and *Salmonella enterica* serovar Typhimurium (*S*. Typhimurium)^27,28^, as well as parasites, such as *Cryptosporidium parvum* (*C. parvum)*^29,30^, viruses^31–33^, and toxins/drugs^32,34,35^ has proven instrumental for understanding the initial steps of pathogenesis and establishing organoids as an advanced model for studying host–pathogen interactions. Microinjection was also used to experimentally test the hypothesis that pks-positive *E. coli* contribute to colon carcinogenesis; for this, long-term exposure to the bacteria was necessary. However, the primary cells constantly regenerate and thus need biweekly splitting. Therefore, the group microinjected the same culture repeatedly over a period of 5 months. Whole-genome sequencing then showed that the infected primary cells had developed a specific mutational signature that has also been identified in colon cancer, thus linking bacterial infection with carcinogenesis^36^.

Moreover, microinjection can also be used to compare the impact of infection from the apical versus the basolateral side. For example, it has been a longstanding hypothesis that gastrointestinal epithelial cells mount differential responses to infection from the basal or apical side to discriminate between microbial stimuli normally present in the lumen of the gut and a potentially life-threatening attack by a pathogen that has managed to break through the epithelial barrier. The microinjection of a virus to either the basal or apical side of colon organoids indeed resulted in such an asymmetric immune response^32^. In contrast, the bacterial cell wall component lipopolysaccharide (LPS) was able to provoke a similar response from both sides^35^.

Despite its advantages, microinjection can present some technical limitations. For instance, different numbers of cells per organoid within the same culture makes the multiplicity of infection difficult to control. Because organoids also have different diameters, not all of them can be injected with the same ease. Moreover, this method is relatively labor intensive. In our experience, a well-trained researcher can microinject 100–200 human gastrointestinal organoids in 5–10 min, injecting the 30–50 largest organoids per well in a 4-well plate. To enable high-throughput processes for the study of gastrointestinal microbiota using colon organoids, an organoid microinjection platform was developed. Aided by computer vision, 90 organoids were injected per hour with bacterial cultures, and luminal content from the organoids could also be retrieved for further analysis^37^. It should be noted that at least gastrointestinal organoids are not static and naturally exhibit rotation, swelling, and inflation^38^.

#### Addition to the medium: Basal- and apical-out organoids

One alternative to microinjection is the addition of pathogens or compounds directly to the culture medium (Fig. 3a). This technique has been widely used to treat organoids with cytokines or bacterial toxins, such as TNF-ɑ and IFN-γ^39^, Shiga toxin^40^, and *C. difficile* toxin A and B^41–44^. One drawback of this method is that the drug or substance has to diffuse through the ECM, and thus organoids located at the center of the ECM dome are not as accessible as those located at the edges. Furthermore, stimulation occurs only on the basolateral side, whereas receptors stimulated by pathogens or their toxins may be located only on the apical side.

To gain better access to the basolateral side, organoids can be released by incubation with cold PBS or other recovery solutions that break down the ECM. This keeps the organoid structure intact, while having the organoids in suspension solves the issue of poor and uncontrolled matrix diffusion. This technique has been applied in the study of bacterial and viral infection of organoids^45–48^. For example, it was also used to infect iPSC-derived cerebral organoids with Zika virus. This virus is particularly harmful to the developing brain; thus, iPSC-derived cerebral organoids, resembling the human brain in early development, are a very good model. Infected brain organoids showed disrupted cortical layers and reduced proliferative zones, recapitulating the Zika-induced microcephaly observed in vivo^49,50^.

As the addition of the infectious agent to the medium exclusively exposes the basolateral side of organoid cells to infection or stimulation, this method is thus particularly useful when the effect of basal contact is the subject of interest.

To expose the apical side of organoids, the apical-out organoids developed by the lab of Manuel Amieva are also practical (Fig. 3a). Removing the ECM and incubating organoids in low binding culture plates leads to reversal of the organoid polarity after a few days while maintaining the barrier integrity and functional characteristics of the organoid culture^51^. This method has also been used in the study of *S*. Typhimurium^51^ and SARS-CoV-2 infection^52,53^. The host changes that actually lead to the invagination-movement of the organoids are currently enigmatic, and there is a potential risk that the removal of ECM may induce an (unknown) response in the host cells. Nevertheless, the method is very attractive because it allows apical infection in 3D without the labor-intensive use of microinjectors for infection or treatment.

#### Infection during splitting

One of the simplest methods to infect organoids is to do so during passaging. After release from the ECM, organoids are disrupted mechanically or enzymatically, shredded into fragments, incubated with the pathogen, and re-embedded in the ECM until the end of the infection time point (Fig. 3a). As with normal passaging, the individual organoid fragments will generate new organoids. This method has been used to infect fallopian tube organoids with *Chlamydia trachomatis* (*C. trachomatis*), an intracellular pathogen. Interestingly, the bacteria did not infect all cells within the organoids but instead established a chronic infection of only a few cells per organoid. The infection could be maintained in vitro for up to 2 months, sustained only by repeated pathogen life cycles within the organoids themselves. This mimics the natural process very closely and enabled the observation that epithelial cells organized into an intact layer can maintain the integrity of this layer by expelling infected cells into the lumen before they are lysed. These observations highlight the advantages of organoids compared to cancer cell lines, in which *C. trachomatis* infection leads to the death of all cells by the end of the first *C. trachomatis* life cycle. Such long-term infections may also be instrumental to study the contribution of this pathogen to cancer development^54^. The same approach was also applied to analyze the correlation between *S*. Typhimurium infection and gallbladder cancer. Infected single cells derived from gallbladder organoids displayed a higher level of organoid formation and cell transformation, which correlates with the known carcinogenic potential of this pathogen^55^.

One aspect that should be considered with this method is that cells forming new organoids are usually stem cells or early progenitors^9^. Therefore, if an infectious agent targets differentiated cells, it is possible that although the cells are infected in the splitting process, the newly formed organoids after reseeding may not contain any infected cells simply because they only originate from (noninfected) stem cells. Additionally, as with inside-out organoids, ECM removal and singularization may trigger responses in host cells.

### Organoid-derived monolayers

Organoid-derived monolayers are established by the enzymatic dissociation of 3D organoids into organoid fragments or single cells that are then seeded on a flat plastic surface or transwells (Fig. 3b). Cell attachment to the surface often requires coating with ECM-like proteins, such as complex matrices (e.g., Matrigel or Basement Membrane Extract), gelatin, collagen, or fibronectin. Complex matrices provide a suitable coating surface but have several disadvantages, such as xenogeneic origin, the presence of growth factors and other components such as gentamicin, and batch-to-batch variability. Collagen is often a good alternative to support the attachment and growth of several types of organoids (e.g., stomach and intestinal organoids)^35,56–58^. To obtain a confluent, stable monolayer of cells attached to the well surface, several experimental aspects must be considered. For example, the number of cells seeded will determine the time required to reach confluence. The medium composition can also be modified to induce or inhibit cell differentiation, depending on the experimental approach. 2D monolayers have been used to model a range of infections, including *H. pylori*^56,59,60^, pathogenic *E. coli*^61^, norovirus^57,62^ and, recently, Epstein Barr virus (EBV) infections^33^. In the case of EBV, infected B cells, the primary target cell of the virus, were added to the epithelial culture and transferred the virus to cells^33^, an infection method termed “transfer infection” that has been previously described for cell lines^63^.

There are several practical advantages of organoid-derived monolayers. Monolayers provide access to the apical side of cells without the need for special equipment and allow the addition of pathogens to cells and removal of pathogens and/or supernatant, cell debris, and waste products to be easily achieved. Because monolayers are usually flat, analysis by microscopy is more straightforward and can be implemented in high-throughput screening. However, certain characteristics, such as cell differentiation and intercellular connections, can differ between organoids and organoid-derived monolayers, and this is an important aspect to consider when choosing an infection model. For example, despite having the potential to generate different cell types from the tissue, especially under specific differentiation conditions, organoid cultures under expansion conditions often contain primarily undifferentiated cells with high proliferative activity. This can impact infection. For example, the replication of norovirus in organoids was a breakthrough in the field because previously, this virus could not be cultured in vitro. However, replication has only been reported in differentiated enterocytes from intestinal organoid-derived monolayers^57,62^, while to our knowledge, reports of such a successful infection in 3D organoids are lacking, possibly because they differ in their state of differentiation. Last, protocols to generate organoid-derived monolayers differ among labs, for example, regarding the coating material, seeding procedure, time allowed to reach confluence, and media composition. It is likely that this affects cellular differentiation and possibly also reproducibility among labs.

To provide access to both sides of the epithelium in monolayer cultures, monolayers can be grown in transwell or other permeable inserts (Fig. 3b). This allows cells to secrete and take up molecules from both cellular surfaces, enabling more physiological metabolic activity. In contrast to monolayer or organoid cultures, transwell cultures enables better assessment of the classical functionality of epithelial barrier polarization and integrity, e.g., by permeability and transcytosis assays^64^. In addition, polarization of the monolayer can be quantified by directly measuring the transepithelial electrical resistance. Since transwells often support cultures for longer periods, cell differentiation and polarization can be induced for a longer time, allowing higher levels of differentiation to be reached. For instance, the presence of microfold (M) cells in intestinal organoid-derived transwell cultures enabled the analysis of *Shigella flexneri* (*S. flexneri*) infection, as it is the preferred cell type for bacterial invasion^65^. Infection from both the apical and basal compartments showed that the bacteria preferentially invaded the basolateral side, leading to greater disruption of the epithelial barrier^65^. Stimulation of both apical and basolateral cellular receptors has also demonstrated an asymmetrical immune response to viral infection^32^. Similar to 3D organoids, the directed differentiation of monolayers can be further induced by the stimulation or inhibition of certain cellular pathways, such as the WNT pathway. For example, the withdrawal of WNT and R-spondin from the culture medium induced a dramatic increase in the SARS-CoV-2 entry receptor ACE2 on the surface of intestinal organoid-derived enterocytes, which in turn enhanced viral infection^66^.

Given that epithelial cells encounter different environmental conditions on their apical and basolateral sides in vivo, mimicking those conditions in the transwell system can induce even further cell differentiation. For example, bladder stem cell progenitors seeded on transwells can form a stratified bladder epithelium that differentiates only after the apical addition of urine. Importantly, this is the first in vitro model of terminally differentiated bladder epithelial cells that tolerate long-term culture and support the intracellular replication of the uropathogen *Enterococcus faecalis*^67^.

Furthermore, transwells allow the exposure of cells to an air–liquid interface (ALI; Fig. 3b). In an ALI model, cells are in contact with the cell culture medium only from the basolateral side, whereas the apical surface is exposed to air. This model has shown that air exposure induces higher levels of cell differentiation^31,60,68,69^. For example, gastric organoids grown in an ALI model can generate long-lived, highly polarized columnar epithelial monolayers, which do not require passaging. Long-term differentiation allows the cells to differentiate fully and produce a thick, protective layer of gastric mucus, hence the term “mucosoids”, resembling the gastric epithelium^60^. In addition, this method enables chronic infection with *H. pylori* for up to 4 weeks. Given that normal cell lines can support *H. pylori* infection for only a few hours, this represents a major advantage. Similarly, gallbladder organoid-derived mucosoids can survive *Salmonella enterica* serovar Paratyphi (*S*. Paratyphi) infection for 7 days^69^.

Overall, transwell and ALI cultures are helpful to assess the apical and basal sides simultaneously and may increase cellular differentiation and even allow long-term culture in some organ models. Limitations of this method include the fact that the currently used membranes do not allow light microscopy and that the inserts are difficult to scale for high-throughput or microscopy-based screening.

As illustrated here, many methods and techniques have been described and are now available for studying infection in organoid and organoid-based models. When choosing a method to answer a specific scientific question, several aspects should be considered. Which model contains the target cell type of the pathogen? Can this cell type be enriched under certain conditions? Does the pathogen prefer apical or basolateral interaction? Does the experiment need a high-throughput readout? Should the model support the infection long-term? Last, the closed lumen of a 3D structure will also provide a different environment than an open 2D layer, for example, regarding oxygen levels and the accumulation of mucus, cell debris, or metabolites. How might this environment impact the infection of interest? These thoughts should be the basis for the selection of a method.

### Tissue engineering models

The term TE was described for the first time by Langer and Vacanti in the 1990s as an “interdisciplinary field which applies the principles of engineering and life sciences toward the development of biological substitutes that restore, maintain, or improve tissue function”^70^. Since then, TE models, including ECM-embedded scaffolds, bioreactors, and organs-on-a-chip, have been developed as reliable tools for modeling healthy or diseased organs to be used for drug screening and the development of new therapies (Fig. 3c). Such systems are becoming increasingly sophisticated, mimicking complex features of tissues and organs, and are now also used for infectious disease research. Although in most cases, these systems have utilized cell lines as biological materials, recent work has shown that using cells from organoids can provide a powerful tool to study pathogenesis in a more physiologically relevant environment. Since organoids can generate most of the different cell types of the tissue from which they were isolated, they can considerably increase the complexity of such models^71,72^. TE techniques can be classified into different types.

#### Cells seeded on scaffolds

A wide range of biomaterial scaffolds has been used as support for cell cultures, which include decellularized matrices, hydrogels, and natural-based or synthetic biopolymers with tunable mechanical and chemical properties (Fig. 3c). The use of biological decellularized scaffolds can help to mimic the 3D structure of tissue by providing the natural architecture for cells^73^, e.g., the widely used small intestinal submucosa (SISmuc) scaffold^74^. This model consists of a piece of decellularized porcine SISmuc. Seeding this scaffold with Caco-2 colorectal cells under dynamic conditions (on an orbital shaker) yields a polarized mucosal epithelial barrier with a 3D microarchitecture resembling that of the small intestine^75^. Infection of this 3D model compared to a standard 2D monolayer of Caco-2 cells with a range of *Campylobacter jejuni* (*C. jejuni*) mutants revealed bacterial factors that play a role in the infection in 3D but not in 2D. For example, *C. jejuni* strains lacking the capsule surrounding the bacteria can adhere and internalize better into host cells, but this effect is only observed in the 3D model^75^. It will be interesting to analyze which molecular mechanisms underlie such effects. By seeding primary organoid-derived cells onto SISmuc, an even more relevant infection model has been generated^64^. In another version, Caco-2 cells were again used to build an epithelial layer on SISmuc to generate a trilayered model (endothelium, scaffold, and epithelium), in which a layer of endothelial cells was seeded onto the other side of the scaffold. When this model was cocultured with monocytes on the endothelial compartment and infected with *S*. Typhimurium on the side seeded with Caco-2 cells, the bacteria mainly infected the epithelial cells, while the endothelial cells were largely protected^76^. A similar decellularized intestinal matrix, but of human origin, has also been used as a scaffold for seeding primary human jejunal organoids derived from pediatric patients. This yielded jejunal grafts that showed not only cell differentiation but also digestion and absorption functions in vitro and could further survive and form intestinal lumens in vivo when grafted under the kidney capsule in mice^77^.

To avoid the decellularization process and dependence on biological tissue, artificial scaffolds can be designed and built to mimic tissue structures and then coated with hydrogels or other types of ECM-like proteins to improve their functionality^78^. Such engineered scaffolds seeded with primary cells from organoids will be a highly valuable tool for future research because they combine the advantages of 3D models, closely mimicking the in vivo situation, with an open surface that is easily accessible for infection experiments. A disadvantage of biological matrices is that they depend on additional primary material. Additionally, seeding of the scaffolds requires large numbers of primary cells, and although expanding organoids in sufficient numbers is technically feasible, it takes time and is costly. Another drawback is that (live) microscopy is restricted by the thickness of the tissue.

#### Bioreactors

A bioreactor is defined as a device or system that supports a biologically active environment, enhancing cell metabolism and growth with continuous nutrient and oxygen circulation, e.g., the frequently used rotating wall vessel (RWV) bioreactor. This bioreactor maintains cells in an optimized suspension culture, enabling the formation of self-organizing 3D tissue-like aggregates. Here, cells can be combined with scaffolds and loaded into the RWV, allowing autocrine and paracrine communication in an effort to mimic the tissue’s natural microenvironment. The RWV was the first technology used to develop 3D models for infection studies with bacterial pathogens and cell lines, but not (yet) organoids^79^.

One particular type of bioreactor, the spinning bioreactor, was instrumental for analyzing the impact of Zika virus on the developing forebrain. In the relatively large cerebral organoids generated from iPS cells, nutrient and oxygen absorption are crucial to facilitate long-term survival and growth. Bioreactors enable longer survival and thereby also differentiation toward more specialized cell types and regions of the brain. Zika virus infection in forebrain-specific organoids generated in a spinning bioreactor led to underdeveloped organoids, with features resembling those of microencephaly, the major pathology induced by the infection in human fetuses^80^.

#### Bioprinting with cells

Hydrogels are highly tunable, and their biofunctionality can be improved through modification of their polymeric network. Moreover, their versatility allows combination with several TE techniques for scaffold production, such as micropatterning, 3D printing, and microfluidic techniques. By micropatterning, it is possible to stamp ECM proteins (e.g., fibronectin and collagen) onto culture substrates (e.g., polystyrene), creating small cell adhesion sites that can mimic the natural scaffold of the tissue. Using cells from organoids in this way has allowed the generation of an in vitro self-renewing human intestinal epithelium with crypt-villus structures and proper cell lineage compartmentalization^81^. Another recent work has described a human intestinal model created using 3D printing and a cell-laden collagen bioink^82^. Similar approaches have also been implemented for other organs, such as the liver^83^. Although conventional cell lines were used in these models, similar tissue-like structures are likely to develop using organoid-derived cells.

#### Organs-on-a-chip and microdevices

In this approach, cells are seeded into a chamber and subjected to continuous flow, with additional mechanical forces that mimic physiological organ conditions (Fig. 3c). These forces provide further complexity to the cultures and mimic the hydrostatic forces to which tissues are normally subjected. Some of these devices include multiple chambers separated by a porous membrane, which can be seeded with different cell types on each side. The chambers can also be seeded with cells from different organs (multiorgan devices) in an attempt to mimic an entire or partial body system (body-on-a-chip)^84,85^. A broad range of organ-on-a-chip models have been developed over recent years, including those models of the skin, lung, gastrointestinal tract, kidney, and liver^86^. Prime examples of organ-on-a-chip models for infection research are those of the lung and intestine.

One such model of airway epithelium used microfluidic devices in combination with primary, tissue-derived, human adult airway cells grown on an ALI platform^87^. The device was composed of a multicompartmental construct with three vertically stacked culture chambers, matching the microarchitecture of the airway mucosa. This enabled physical separation between the different airway cell types and allowed paracrine signaling across the nanoporous membranes. However, most lung-on-a-chip models used for infection studies so far have relied on monotypic cancer cell lines, e.g., to assess the expression of surfactant protein A in response to *Staphylococcus aureus* and influenza A infection^88^, but future chip models incorporating primary cells from organoids are expected.

Similarly, the human gastrointestinal tract has been modeled using a gut-on-a-chip microdevice to obtain a more physiologically relevant model of the intestine. While most models currently rely on cancer cell lines as biological material, these studies lay a foundation for future, more sophisticated models using organoids. One such microdevices was designed to allow movement of the membrane on which the cells were seeded: by changing the vacuum in neighboring channels, the membrane could be rhythmically strained and stretched^89^. The same device was later employed to determine the impact of mechanical forces on *S. flexneri* infection^90^.

Efforts are also being made to combine the TE models described above with organ-on-a-chip devices. For example, Caco-2 cells were combined with an endothelial cell layer in a gut-on-a-chip model. The addition of macrophages and dendritic cells on top allowed the formation of a more immunocompetent environment that could be applied to study the effect of the presence of the microbiont *Lactobacillus rhamnosus* on infection with the pathogen *Candida albicans*^91^.

Additionally, several labs have combined 3D architectures resembling crypts or crypt-villus structures with organ-on-a-chip devices. An early version used Caco-2 cells and could support the growth of one bacterial strain from the microbiota^92^. A different microfluidic model of the gut, named HuMiX, composed of three microchambers, has also applied in the study of human-microbial crosstalk and could sustain the growth of the obligate anaerobic bacteria *Bacteroides caccae*^93^. An updated version of this device, seeded with intestinal organoid-derived cells, can sustain a complex microbiota, further highlighting the increased physiological complexity that can be achieved with primary cells. In particular, using a microfluidic intestine-on-a-chip with a transluminal hypoxia gradient, the growth of over 200 unique taxonomic units could be achieved, including obligate anaerobic bacteria, to a similar level as that found in human stools^94^. This device has now also been used by other research groups to study host–pathogen interactions with human intestinal epithelia, for example, by treating the epithelium with the heat-stable enterotoxin A of enterotoxigenic *E. coli*^95^. Recently, a mini-intestine model consisting of a hydrogel-coated microdevice seeded with intestinal organoid-derived cells has been reported. The resulting tube-shaped epithelium has an accessible lumen that mimics the spatial arrangement of crypt- and villus-like domains and can support the whole infection cycle of *C. parvum*^96^.

Such models with flow overcome one very simple limitation of in vitro infection studies: once bacteria are added to any culture, 3D or 2D, microorganisms will feed on the nutrients in the medium provided to nurture the cells, and many bacteria, such as *E. coli*, will overgrow and kill the host cultures within hours. Although inhibition by antibiotics is frequently used to delay this, it is far from ideal. In an open tube that is washed by the flow of the medium either continuously or in frequent pulses, bacteria are regularly removed. Therefore, this study highlights the vast potential of the combination of scaffolds, organ-on-a-chip devices and organoids: this is the combination of a 3D structure with growth factor gradients and fluidics that not only stimulates primary cells to self-organize and reach homeostasis but also inhibits the overgrowth of an infection. Future devices will further advance this principle.

## Outlook/Concluding remarks

Organoids have dramatically advanced the field of infection research, contributing important findings to a better understanding of host–pathogen interactions and disease pathogenesis. Organoids are tractable models that allow the investigation of cellular and molecular pathways triggered by invading pathogens while maintaining some of the architecture of the original tissue. Future challenges to the field relate to the incorporation of further complexity, namely, the presence of immune and nerve cells, vasculature, and even the microbiome^97–100^. Advances in these areas include the development of several approaches for coculturing immune cells with epithelial organoids^97^, e.g., the coculture of neutrophils with an RSV-infected airway organoid^31^, coculture with B cells for transfer infection with EBV^33^, or coculture with monocytes in an *S*. Typhimurium-infected tissue engineered intestinal model^76^. Regarding the incorporation of nerve cells, a human intestinal organoid containing a functional enteric neuroglial plexus has been described^101^. While the presence of a mucus layer in organoids is a great advantage compared to standard cancer cell lines, achieving more complex mucus layers would be an important advancement in the future.

Organ-on-chip devices can recapitulate vascular flow, tissue–tissue interactions, and organ-relevant mechanical movements. Most of organ-on-a-chip models applied to study infection processes have been fabricated using epithelial cell lines. Combining these models with organoid cultures and integrating other TE approaches, such as including a tissue architecture, will increase the complexity of the epithelia and mimic the tissue even more closely. In addition, organ-on-a-chip technology can combine organoids from several different organs (such as the liver and intestine connected via microfluidics), adding yet another level of complexity, ultimately aiming to model in vitro the physiology of whole organisms in the future.

All these aspects are key determinants of infection, and their incorporation into organoid-based models will transform these techniques into more physiological systems. This will in turn complement in vivo experimentation and possibly allow a reduction in animal experiments. The continued development of organoids into ever more sophisticated research models is strategically important in response to the pressure to move away from animal experimentation for ethical reasons. Initiatives to develop such strategic methods have so far focused primarily on toxicology testing, which is a completely different context in both that it is heavily standardized and that the methodological choices are strongly determined by regulations. The requirements of biomedical research are different, and the transition to non-animal-based methods will depend on these models proving to be valid and relevant for translational research.

Human stem cell-based organoids thus hold great potential in this context and have already provided some highly translatable insights, for example, in drug testing for cystic fibrosis. Regarding translation, it should be noted that an increase in complexity per se is not necessarily always an improvement because drug testing platforms need scalable, reproducible, and very clear readouts, which can mean a reduction in complexity. Thus, it can be envisaged that rather simple, organoid-based assays in 3D or even 2D will help to identify new therapeutic approaches to combat infection in the future. More complex models incorporating some of the abovementioned advances to more closely mimic tissues will then allow more in-depth study of the mode of action of new therapeutics.

Last, as organoids can be generated potentially from every patient, they also hold promise to allow insights into patient-specific responses in the future, which will not only allow an understanding of why certain patients react differently to an infection to be gained but also hopefully pave the way for the identification of risk factors and patient-specific therapies.
